# Serum leptin level is associated with phase angle in CKD5 patients not undergoing dialysis

**DOI:** 10.1371/journal.pone.0202055

**Published:** 2018-08-08

**Authors:** Jun Young Lee, Jae-Seok Kim, Jae-Won Yang, Seung Ok Choi, Joon Hyung Sohn, Byoung-Geun Han

**Affiliations:** 1 Department of nephrology, Yonsei University Wonju College of Medicine, Wonju, Kang-won, Korea; 2 Department of Biochemistry and Institute of Basic Medical Science, Yonsei University Wonju College of Medicine, Wonju, Kang-won, Korea; University of Liège, BELGIUM

## Abstract

**Objective:**

Malnutrition is very complex in patients with end-stage renal disease (ESRD) and is associated with poor prognosis. This is because hemodynamic changes, hormonal changes, persistent inflammatory reactions, and fluid overloads are more complicated as uremia is worsening. Bio-impedance spectroscopy (BIS) is a useful method to estimate fluid balance (Overhydration/ extracellular water, OH/ECW) and nutritional status (Phase angle, PhA). We aimed to evaluate the volume and nutritional status by BIS and to investigate the relationship between the appetite regulating hormones and the parameters of BIS in patients with stage 5 chronic kidney disease not undergoing dialysis (CKD5-ND).

**Methods:**

We enrolled a total of 91 CKD5-ND patients. We measured routine serum markers including albumin and NT-proBNP and the appetite regulating hormones, leptin and ghrelin. We defined poor nutritional status as a PhA < 4.5°, and proper nutritional status as a PhA ≥ 4.5°. We also evaluated each patient’s nutritional status by assessing their geriatric nutritional risk index (GNRI) and their volume status by measuring NT-proBNP.

**Results:**

Forty-one patients (45%) had poor nutritional status. Patients with a poor nutritional status had significantly higher OH/ECW (29.6 ± 12.7% vs. 6.2 ± 10.3%, p<0.001) and lower levels of leptin (3.8 ± 3.1 vs. 7.0 ± 6.2 ng/mL, p = 0.004) than those with proper nutritional status. PhA was associated with GNRI (r = 0.597, P<0.001) and NT-proBNP was associated with OH/ECW (r = 0.384, P<0.001). Leptin was negatively correlated with OH/ECW (r = -0.288, p = 0.006). In contrast, leptin was positively correlated with PhA (r = 0.263, p = 0.012). In multivariate logistic regression, high level of leptin (OR 7.00, 95% CI 1.74–28.10) was associated with proper nutrition, while an increased OH/ECW (OR 0.65, 95% CI 0.51–0.84) was associated with poor nutrition.

**Conclusions:**

Our study demonstrates that CKD5-ND patients with poor nutrition generally also suffer from excessive body fluid. Low leptin level suggests poor nutrition in CKD5-ND patients. PhA could be used as a nutritional index for ESRD patients.

## Introduction

Malnutrition is a common problem in patients with end stage renal disease (ESRD) [1] and is associated with a higher rate of mortality in this population [2]. Stage 5 chronic kidney disease not undergoing dialysis (CKD5-ND) are mostly overhydrated [3], which is associated with malnutrition and exacerbates the malnutrition, inflammation and atherosclerosis complex [4]. Therefore, when evaluating the nutritional status of CKD5-ND patients, it is helpful to evaluate the volume status of the patients. Dual energy X-ray absorption spectrometry (DEXA) and bio-impedance spectroscopy (BIS) can both simultaneously evaluate nutritional status and the volume status of patients. DEXA, however, is expensive and requires use of specialized instrumentation, limiting its practical use. In contrast, BIS is an easy, accurate, and non-invasive method that can be used to simultaneously assess the nutritional status and volume status of a subject [5–8].

In particular, phase angle (PhA), which is one of the BIS parameters, reflects nutritional status well. PhA is the arctangent of the ratio of reactance (Xc) to resistance (R) measured by current flow [9]. R is the restriction to the flow of an electric current and is primarily related to the amount of water present in the tissues. Xc is the opposition to a change in voltage due to the element’s capacitance. It represents the ability of tissues to store energy. Because the cells have a similar electrical capacity, the greater the number of cells, the larger the reactance. R represents body water content and Xc is related to body cell mass. Theoretically, PhA can be used as a nutritional indicator, because malnutrition is characterized by alterations in fluid balance and changes in cellular membrane integrity [10]. In practice, PhA has been widely used as a nutritional assessment tool in patients with liver cirrhosis, colon cancer, and ESRD [7,9,11]. These studies we mentioned have reported that decreased PhA is correlated with poor nutritional status and prognosis in ESRD, but no studies have been conducted in CKD5-ND patients.

Loss of appetite is a major and correctable cause of malnutrition in chronic kidney disease (CKD) [12]. Appetite is regulated by orexigenic and anorexigenic signals originating from the hypothalamus. Leptin and ghrelin are known to play important roles in regulating appetite control signals [13]. The association between nutritional status and appetite regulating hormones such as leptin and ghrelin in CKD5-ND patients, however, is unknown. We aimed to evaluate the volume and nutritional status by BIS and to investigate the relationship between the appetite regulating hormones and the parameters of BIS in CKD5-ND patients.

## Materials and methods

### 1. Patients and data collection

This study included one hundred four patients who visited our hospital between October 2014 and May 2016 to plan either hemodialysis or peritoneal dialysis for renal replacement therapy. We retrospectively analyzed BIS data for this cohort of patients obtained at the planning visit. Underlying disease and demographic characteristics were investigated. The laboratory study, echocardiography, and BIS were performed before the first dialysis session. Patients with malignancy (N = 2), liver cirrhosis (N = 1), low left ventricular ejection fraction (N = 1), urgent dialysis before BIS analysis (N = 1), and acute kidney disease (N = 8) were excluded. Ninety-one patients were finally enrolled in this study. This study was approved by the Institutional Review Board of Yonsei University Wonju Severance Christian Hospital. All participants provided written informed consent prior to the study.

### 2. Assessment of volume and nutritional status

Before starting renal replacement therapy, BIS was performed with BCM^™^ (Body Composition Monitoring^™^, Fresenius Medical Care, Bad Homburg, Germany) to assess the volume and nutritional status of each patient. BCM^™^ measures intracellular water (ICW), extracellular water (ECW), and total body water (TBW) by sending currents with 50 different frequencies from 5 to 1000 kHz into the body and measuring each current’s impedance. It estimates volume status by reporting the overhydration (OH) value. BCM^™^ also reports the fat tissue index (FTI), lean tissue index (LTI), and PhA to evaluate the patients’ nutritional status. PhA is an angle value of the time delay between the voltage waveform at 50 kHz and current waveform. The validity of BIS in healthy individuals and ESRD patients in comparison to standard measurement methods has been demonstrated in previous studies [5,14]. We assigned patients with a PhA less than 4.5° (PhA < 4.5°) to the malnutrition group, and those with a PhA above 4.5° (PhA ≥ 4.5°) to the normal nutrition group [7]. To reduce bias due to the BCM^™^ machine, we also calculated the geriatric nutritional risk index (GNRI) [15] to determine nutritional status and measured N-terminal prohormone of brain natriuretic peptide (NT-proBNP) [16] to determine volume status. GNRI was calculated using height (H: cm), body weight (BW: kg), and serum albumin level. First, ideal weight (WLo: kg) was calculated according to gender from the Lorentz equations as follows:

Men;WLo=H−100−[(H−150)/4],Women:WLo=H−100−[(H−150)/2.5]

Then albumin, ideal weight, and body weight were substituted into the following formula

GNRI=[1.489×albumin(g/L)]+[41.7×(BW/WLo)]

(BW/WLo = 1, when BW exceeded WLo).

### 3. Laboratory and echocardiographic evaluations

Serum NT-proBNP was measured by electro-chemiluminescence immunoassay (ECLIA) on a Modular Analytics E170 clinical analyser (Roche Diagnostics, Mannheim, Germany). Analytical measurement range for NT-proBNP was 5 to 35,000 pg/mL. All blood samples were collected before initiation of renal replacement therapy and were then immediately centrifuged and stored at -73°C until analysis. Serum leptin and ghrelin levels were measured with enzyme-linked immunosorbent assay (ELISA) kits. Samples were assayed for leptin (ELISA kit for leptin, Cat. No. SEA084Hu; Cloud-Clone, TX, USA) and ghrelin (ELISA kit for ghrelin, Cat. No. CEA991Hu; Cloud-Clone, TX, USA) in duplicates and the mean value of the two measures was used in the analysis. Analytical measurement range for leptin and ghrelin were 0.156 to 10 ng/mL and 123.5pg/mL to 10,000 pg/mL, respectively. Reference distributions of this leptin and ghrelin are 2.2–8.6 ng/mL and 161–856 pg/mL in healthy people. Glomerular filtration rate (GFR) was calculated by modification of diet in renal disease (MDRD) equations. Echocardiography was performed all patients by using 3-MHz transducer and commercial ultrasound system (Vivid-7, General Electric-Vingmed, Milwaukee, WI, USA).

### 4. Statistical analysis

All statistical analyses and graphs were performed using with IBM Statistics Package for the Social Science (SPSS) version 23.0 (IBM Corporation, Armonk, NY, USA). Categorical data were described as frequencies and percentages. Descriptive statistics were described as means ± standard deviation (SD) for continuous variables. Unpaired Student’s t-test was used to determine the significance of differences in clinical variables between the two groups. The chi-square test was used to compare categorical variables. Pearson’s correlation test was used to examine relationships between variables. Multivariate logistic regression was performed using leptin, albumin, and OH/ECW. These variables were chosen considering collinearity among the factors that showed a statistically significant correlation with PhA. Odds ratios (OR), 95% confidence intervals (CI), and p-values are reported. Because there is no definite reference value of leptin, we categorized leptin level into tertiles according to percentiles (<2.59ng/dL, 2.59–6.05ng/dL, >6.05ng/dL). OR was calculated using the lowest tertile as the reference. In model 1, we analysed the variables after adjusting for age and sex. In model 2, we analyzed variables after adjusting for age, sex, C-reactive protein (CRP), creatinine (Cr), and LTI. In model 3, we analyzed variables after adjusting for age, sex, CRP, Cr, LTI, presence of diabetes, use of diuretics, and presence of nephrotic syndrome. Goodness-of-fit of the model was assessed using the Hosmer-Lemeshow test. Predictive accuracy of each logistic regression model was evaluated by calculating the c-statistic (equivalent to the area under the receiver operating characteristic curve). Statistical significance was defined as P<0.05.

## Results

### 1. Characteristics of patients

Mean age of patients was 59.77 ± 11.19 (range 31–79) years. Fifty patients were male. Mean PhA and ECW were significantly higher in males (4.66 ± 1.31°, 19.1 ± 4.8 L) than in females (3.97 ± 1.20°, 15.6 ± 5.1 L)(P<0.05). Other variables were not significantly different between male and female patients. Fifty-seven patients (62.6%) had a history of diabetes. Diabetic patients had lower mean PhA (4.0 ± 1.24° vs. 4.99 ± 1.17°, P<0.001) and albumin (3.3 ± 0.5 g/dL vs. 3.6 ± 0.7 g/dL, P = 0.010) levels and a higher ECW (18.6 ± 6.0 L vs. 16.0 ± 2.8 L, P = 0.008) level than non-diabetic patients. Other variables were not significantly different between diabetic and non-diabetic patients.

### 2. Differences according to PhA

Normal nutrition group had significantly higher albumin and leptin levels as well as GNRI than the malnutrition group. Normal nutritional group had a lower CRP level than the malnutrition group (0.8 ± 1.3 mg/dL vs. 2.7 ± 4.4 mg/dL, P = 0.017). The difference in ghrelin level between the two groups was marginal (P = 0.052). NT-proBNP, OH, and OH/ECW, all of which reflect volume status, were lower in the normal nutrition group than the malnutrition group (Table 1). In addition, NT-proBNP was associated with OH/ECW (r = 0.384, P<0.001).

**Table 1:** Compare parameters according to nutritional status.

Variables	Total (N = 91)	PhA<4.5° (N = 41)	PhA≥4.5° (N = 50)	P-value
Age (years)	59.8±11.2	60.5±12.8	59.3±9.8	0.656
Gender (male, %)	50 (55%)	20 (49%)	33 (66%)	0.097
HTN (N, %)	81 (89%)	39 (95%)	42 (84%)	0.091
CVD (N%)	26 (28.6%)	12 (29%)	14 (28%)	0.894
Diuretics (N, %)	54 (59.3%)	25 (61%)	29 (58%)	0.774
NSD (N, %)	27 (30%)	15(37%)	12 (25%)	0.212
Leptin (ng/mL)	5.6±5.3	3.8±3.1	7.0±6.2	**0.004**
Ghrelin (pg/dL)	2,104.6±2263.2	1,596.6±2026.9	2,521.3±2379.3	0.052
BMI (kg/ m^2^)	24.9±4.1	24.8±4.4	25.1±3.8	0.662
LTI (kg/ m^2^)	14.3±3.2	13.1±3.5	15.3±2.7	**0.001**
FTI (kg/ m^2^)	9.1±4.5	9.1±3.9	9.2±5.0	0.962
GNRI	91.4±9.3	85.1±7.1	96.5±7.7	**<0.001**
Albumin (g/dL)	3.4±0.6	3.0±0.5	3.7±0.5	**<0.001**
BUN (mg/dL)	91.2±24.8	92.5±26.7	90.2±23.4	0.669
Creatinine (mg/dL)	9.2±3.4	9.4±3.1	9.0±3.6	0.575
CRP (mg/dL)	1.7±3.2	2.7±4.4	0.8±1.3	**0.009**
Hemoglobin (g/dL)	9.2±1.3	9.0±1.5	9.3±1.2	0.409
NT-proBNP (pg/mL)	9,181.0±1667	14,477.8±12712	4,965.2±8824	**<0.001**
OH (L)	3.6±4.5	6.4±5.0	1.2±1.8	**0.000**
OH/ECW (%)	16.7±16.3	29.6±12.7	6.2±10.3	**0.000**
TBW (L)	35.4±8.1	36.2±9.9	34.6±6.2	0.409
ECW (L)	17.7±5.2	19.7±6.4	16.0±3.1	**0.001**
ICW (L)	17.7±3.9	16.5±4.0	18.8±3.5	**0.004**
EF (%)	62.5±0.9	62.9±1.4	62.15±1.1	0.674
GFR (mL/min)	6.6±2.8	6.5±0.4	6.7±2.8	0.738
BP Sys (mmHg)	142.7±19.7	146.0±3.5	140.0±2.5	0.166
BP Dia (mmHg)	78.4±11.6	79.05±1.9	77.9±1.6	0.636

Mean±SD

BMI, Body mass index; BP Dia, diastolic blood pressure; BP Sys, systolic blood pressure; CRP, C-reactive protein; BUN, Blood urea nitrogen; CVD, Cardiovascular disease; ECW, Extracellular water; EF, ejection fraction; FTI, Fat tissue index; GFR, glomerular filtration rate; GNRI, Geriatric nutritional risk index; HTN, Hypertension; ICW, Intracellular water; LTI, Lean tissue index; N, Number; NSD, nephrotic syndrome; NT-proBNP, N-terminal prohormone of brain natriuretic peptide; OH, Overhydration; PhA, Phase angle; TBW, Total body water.

### 3. Correlation between appetite regulating hormones (leptin and ghrelin) and other variables

Ghrelin was positively associated with leptin and BMI and negatively associated with NT-proBNP. PhA, albumin, GNRI, OH, and OH/ECW were not significantly associated with ghrelin. Leptin was positively associated with ghrelin, PhA, BMI, FTI, and GNRI. NT-proBNP, OH, and OH/ECW were negatively associated with leptin. Hemoglobin was not significantly associated with leptin (r = 0.021, P = 0.843), but positively associated with ghrelin (r = 0.234, P = 0.025). LTI, ECW, and ICW, which differed significantly between groups, were not significantly associated with leptin (Table 2).

**Table 2:** Correlation between variables.

Variables	Ghrelin		Leptin	
	r	p-value	r	p-value
Age (years)	0.181	0.087	0.073	0.694
Leptin (ng/mL)	0.238	**0.023**	-	**-**
Ghrelin (pg/dL)	-	-	0.238	**0.023**
PhA (°)	0.107	0.315	0.263	**0.012**
BMI (kg/m^2^)	0.209	**0.047**	0.351	**0.001**
LTI (kg/ m^2^)	0.109	0.304	0.004	0.973
FTI (kg/ m^2^)	0.082	0.430	0.407	**<0.001**
GNRI	0.103	0.332	0.281	**0.007**
Albumin (g/dL)	0.061	0.565	0.205	0.051
NT-proBNP (pg/mL)	-0.273	**0.010**	-0.237	**0.026**
OH (L)	0.039	0.713	-0.239	**0.023**
OH/ECW (%)	-0.13	0.902	-0.288	**0.006**
TBW (L)	0.157	0.138	0.030	0.780
ECW (L)	0.118	0.265	-0.096	0.368
ICW (L)	0.155	0.143	0.065	0.540

r: Correlation coefficient;

BMI, Body mass index; BUN, blood urea nitrogen; ECW, Extracellular water; FTI, Fat tissue index; GNRI, geriatric nutritional risk index; ICW, Intracellular water; LTI, Lean tissue index; NT-proBNP, N-terminal prohormone of brain natriuretic peptide; OH, Overhydration; PhA, Phase angle; TBW, Total body water.

### 4. Predictive factors of nutrition

**Fig 1:**
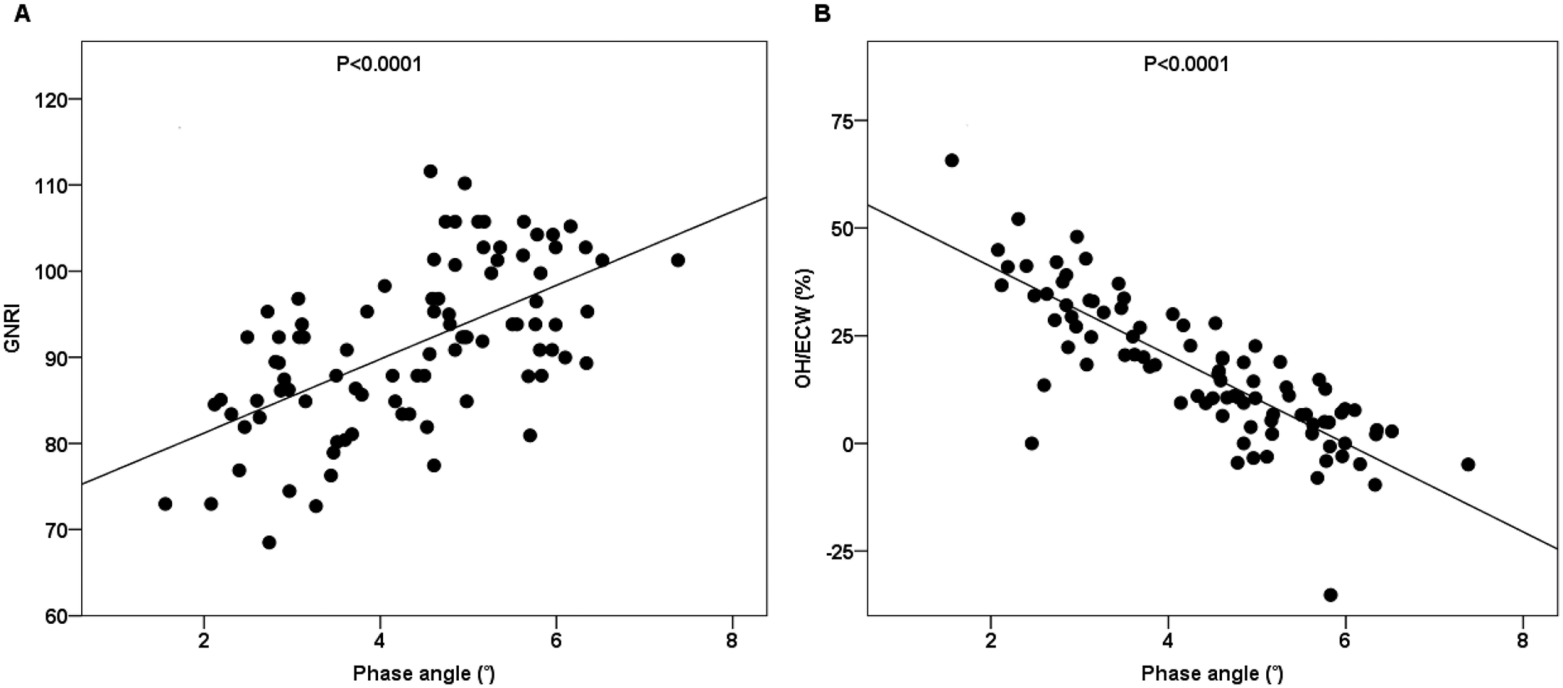
Correlation between phase angle and GNRI (r = 0.597) (A), correlation between phase angle and OH/ECW (r = -0.818) (B).

In Pearson’s correlation analysis, PhA was significantly associated with leptin (r = 0.263, P = 0.012), GNRI (r = 0.597, P<0.001), albumin (r = 0.592, P<0.001), NT-proBNP (r = -0.414, P<0.001), as well as the BIS factors of OH (r = -0.717, P<0.001) and OH/ECW (r = -0.818, P<0.001) (Fig 1). Ghrelin was not significantly associated with PhA (r = 0.107, P = 0.315). PhA was significantly associated with ECW (r = -0.434, P<0.001), ICW (r = 0.383, P<0.001), and LTI (r = 0.387, P = 0.000). Age (r = -0.076, P = 0.472), BMI (r = -0.023, P = 0.825), FTI (r = -0.038, P = 0.725) and TBW (r = -0.098, P = 0.354) were not significantly associated with PhA.

**Fig 2:**
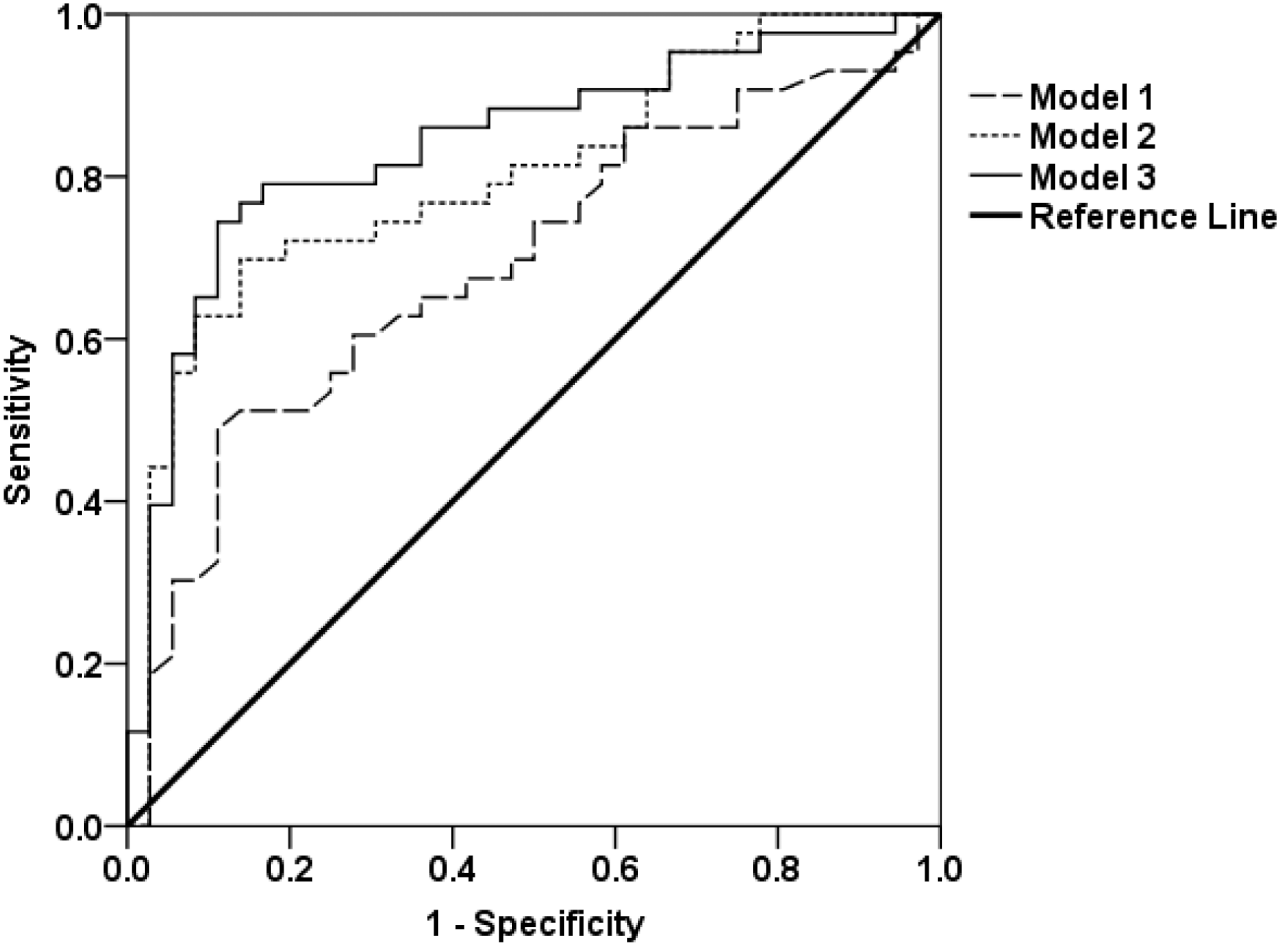
Logistic model prediction accuracy by using c-static.

Univariate logistic regression analysis showed that plasma leptin and albumin levels were positively associated with proper nutrition. In contrast, OH/ECW was negatively associated with proper nutrition. The Hosmer-Lemeshow test showed significant goodness of fit for model 1 (P = 0.505), model 2 (P = 0.905), and model 3 (P = 0.924). The c-statistic was 0.71 (95% CI 0.61–0.82, P = 0.001), 0.79 (95% CI 0.70–0.89, P<0.001), and 0.84 (95% CI 0.75–0.92, P<0.001), for model 1, model 2 and model 3 respectively (Fig 2)(Table 3).

**Table 3:** Logistic regression analysis: Predictive factors of proper nutrition.

Variables	Model 1		Model 2		Model 3	
	OR (95% CI)	p-value	OR (95% CI)	p-value	OR (95% CI)	p-value
Plasma leptin						
Tertile 1 (<2.59ng/mL)	Reference		Reference		Reference	
Tertile 2 (2.59–6.05ng/mL)	2.99 (1.00–8.94)	0.050	3.63 (1.01–13.18)	0.049	4.2 (1.04–17.24)	0.044
Tertile 3 (≥6.05ng/mL)	5.30 (1.68–16.77)	0.005	6.51 (1.71–24.85)	0.006	7.00 (1.74–28.10)	0.006
Albumin (g/dL)	28.67 (6.93–118.59)	<0.001	30.47 (6.74–137.65)	0.017	28.58 (6.08–134.40)	<0.001
OH/ECW (%)	0.77 (0.68–0.86)	<0.001	0.65 (0.51–0.83)	<0.001	0.65 (0.51–0.84)	0.001

Model 1: Adjusted for age and gender

Model 2: Adjusted for age, gender, CRP, creatinine, lean tissue index

Model 3: Adjusted for age, gender, CRP, creatinine, lean tissue index, DM, diuretics, nephrotic syndrome

CI, confidence interval; OR, odds ratio

## Discussion

Generally, leptin passes through the blood brain barrier (BBB) and binds to receptors in the hypothalamus, resulting in appetite suppression due to inhibition of appetite-promoting substances such as neuropeptide Y (NPY), agouti-related protein (AgRP), or direct blocking of the type 4 melanocortin receptor in the hypothalamus [17,18]. Because leptin is predominantly degraded in renal tubules, leptin levels are generally elevated in patients with ESRD [19]. However, it is still under debate whether leptin is a source of anorexia in ESRD patients. Previous studies reported that serum leptin levels were positively correlated with PhA, in hemodialysis patients and with serum albumin in peritoneal dialysis patients [20,21]. A high calorie diet and appetite stimulant administration increased serum leptin levels in patients with ESRD due to an increase in the amount of adipose tissue. However, increased leptin alone may not sufficiently suppress appetite. [22,23]. The ratio of the leptin receptor to leptin was found to be inversely correlated to PhA. While no specific explanations were proposed, this finding suggests that resistance to leptin receptors may affect the nutritional status of patients with ESRD [20]. We found that leptin was positively correlated with nutritional status in ESRD patients. This observed relationship may be because of leptin receptor resistance. Resistance can arise because leptin is inhibited from passing through the BBB and fails to reach leptin receptors [24]. The extracellular leptin binding domain of the leptin receptor also possesses strong homology to the gp130 signal transducing subunit of the receptor for IL-6, an inflammatory cytokine [25]. This structural similarity downregulates leptin receptor signal transduction in a chronic inflammatory state and impairs counter-regulatory processes due to a change in the conformation of the leptin receptor [26]. More specific research on patients with ESRD is needed.

Ghrelin is a hormone secreted mainly from the stomach that promotes secretion of NPY and AgRP in the hypothalamus, thereby increasing appetite [27]. Because ghrelin is mainly degraded in the kidney, its level is increased in ESRD patients [28]. Ghrelin exists in three forms: acyl-ghrelin, des-acyl ghrelin, and obestatin. Acyl-ghrelin increases appetite, while des-acyl ghrelin and obestatin suppress appetite [29]. In ESRD, total ghrelin is increased but the acyl-ghrelin to des-acyl ghrelin ratio changes with nutritional status [30]. This may explain why total ghrelin level was not associated with nutritional indicators in our study. As another report suggested, the acyl-ghrelin to des-acyl ghrelin ratio may be strongly associated with nutritional indicators [31].

Considering the differences according to PhA in albumin and GNRI, which are generally known as indicator of nutritional status, PhA could be used as a nutritional indicator in CKD5-ND. In general, NT-proBNP was increased in patients with heart failure and renal failure. In this study EF and GFR did not make significant difference between two groups, suggesting that poor nutrition group’s NT-proBNP was not increased in by heart failure and renal failure. Hypoalbuminemia, caused by malnutrition, increases vascular permeability and decreases colloid oncotic pressure, leading to a hypervolemic status. In addition, hypervolemic status reduces tissue perfusion, resulting in an inflammatory reaction. In this study, PhA was negative associated with the hydration status indicators. Increased CRP, NT-proBNP, and OH/ECW in malnutrition group means that nutrition, hydration, and inflammation were correlated but we could not determine the causal relationship.

Unlike previous studies, relatively homogeneous group of dialysis naive patients with stage 5 CKD (eGFR < 15 ml/min) were enrolled in our study. It is meaningful considering that the dialysis therapy may also affect the general symptoms and signs of the patients. It was also different from the heterogeneous patient population that includes all stages of CKD patients. Stabilization of volume status and removal of uremic toxin by starting hemodialysis can often improve anorexic symptoms of the CKD patients not undergoing dialysis. Therefore, we thought that nutrition and volume status of advanced CKD patients could be different from dialysis patients.

Our study had several limitations. First, this was a single center study with a relatively small number of patients. Second, the normal range of PhA values for the Korean population has not yet been defined. PhA varies by age, gender, and race [13]. Third, we did not measure the sub-forms of ghrelin, therefore we could not categorize ghrelin into its sub-forms such as the ratio of acyl-ghrelin to des-acyl ghrelin. Fourth, although patients received diet education we did not control patients’ dietary intake (total energy intake and nutrient ratio). Despite these limitations, our study included only patients with stage 5 CKD who had not begun dialysis. We also objectively assessed the volume status of the patients not affecting dialysis therapy at the time of blood sampling using BIS.

Taken together, we suggest that CKD5-ND patients with poor nutrition assessed by PhA and GNRI generally also suffer from excessive body fluid evaluated by OH/ECW and NT-proBNP. Low leptin level suggests poor nutrition in CKD5-ND patients. PhA could be used as a nutritional index for ESRD patients.

## References

[1] Stenvinkel P, Heimburger O, Paultre F, Diczfalusy U, Wang T, Berglund L, et al Strong association between malnutrition, inflammation, and atherosclerosis in chronic renal failure. Kidney Int. 1999;55: 1899–1911. 10.1046/j.1523-1755.1999.00422.x 10231453

[2] Cooper BA, Penne EL, Bartlett LH, Pollock CA. Protein malnutrition and hypoalbuminemia as predictors of vascular events and mortality in ESRD. Am J Kidney Dis. 2004;43: 61–66. 10.1053/j.ajkd.2003.08.045 14712428

[3] Hung SC, Kuo KL, Peng CH, Wu CH, Lien YC, Wang YC, et al Volume overload correlates with cardiovascular risk factors in patients with chronic kidney disease. Kidney Int. 2014;85: 703–709. 10.1038/ki.2013.336 24025647

[4] Kim EJ, Choi MJ, Lee JH, Oh JE, Seo JW, Lee YK, et al Extracellular Fluid/Intracellular Fluid Volume Ratio as a Novel Risk Indicator for All-Cause Mortality and Cardiovascular Disease in Hemodialysis Patients. PLoS One. 2017;12: e0170272 10.1371/journal.pone.0170272 28099511 PMC5242490

[5] Moissl UM, Wabel P, Chamney PW, Bosaeus I, Levin NW, Bosy-Westphal A, et al Body fluid volume determination via body composition spectroscopy in health and disease. Physiol Meas. 2006;27: 921–933. 10.1088/0967-3334/27/9/012 16868355

[6] Mushnick R, Fein PA, Mittman N, Goel N, Chattopadhyay J, Avram MM. Relationship of bioelectrical impedance parameters to nutrition and survival in peritoneal dialysis patients: Management of comorbidities in kidney disease in the 21st century: Anemia and bone disease. Kidney Int. 2003;64: S53–S56. 10.1046/j.1523-1755.64.s87.22.x 14531774

[7] Piccoli A. Identification of operational clues to dry weight prescription in hemodialysis using bioimpedance vector analysis. The Italian Hemodialysis-Bioelectrical Impedance Analysis (HD-BIA) Study Group. Kidney Int. 1998;53: 1036–1043. 10.1111/j.1523-1755.1998.00843.x 9551415

[8] Kim JS, Yang JW, Yoo JS, Choi SO, Han BG. Association between E/e ratio and fluid overload in patients with predialysis chronic kidney disease. PLoS One. 2017;12: e0184764 10.1371/journal.pone.0184764 28902883 PMC5597236

[9] Selberg O, Selberg D. Norms and correlates of bioimpedance phase angle in healthy human subjects, hospitalized patients, and patients with liver cirrhosis. Eur J Appl Physiol. 2002;86: 509–516. 10.1007/s00421-001-0570-4 11944099

[10] Haussinger D, Roth E, Lang F, Gerok W. Cellular hydration state: an important determinant of protein catabolism in health and disease. Lancet. 1993;341: 1330–1332. 10.1016/0140-6736(93)90828-5 8098459

[11] Grundmann O, Yoon SL, Williams JJ. The value of bioelectrical impedance analysis and phase angle in the evaluation of malnutrition and quality of life in cancer patients—a comprehensive review. Eur J Clin Nutr. 2015;69: 1290–1297. 10.1038/ejcn.2015.126 26220573

[12] Pupim LB, Ikizler TA. Uremic malnutrition: new insights into an old problem. Semin Dial. 2003;16: 224–232. 12753685 10.1046/j.1525-139x.2003.16046.x

[13] Mak RH, Cheung W, Cone RD, Marks DL. Orexigenic and anorexigenic mechanisms in the control of nutrition in chronic kidney disease. Pediatr Nephrol. 2005;20: 427–431. 10.1007/s00467-004-1789-1 15662537

[14] Barbosa-Silva MC, Barros AJ, Wang J, Heymsfield SB, Pierson RN Jr. Bioelectrical impedance analysis: population reference values for phase angle by age and sex. Am J Clin Nutr. 2005;82: 49–52. 10.1093/ajcn.82.1.49 16002799

[15] Bouillanne O, Morineau G, Dupont C, Coulombel I, Vincent JP, Nicolis I, et al Geriatric Nutritional Risk Index: a new index for evaluating at-risk elderly medical patients. Am J Clin Nutr. 2005;82: 777–783. 10.1093/ajcn/82.4.777 16210706

[16] Han BG, Song SH, Yoo JS, Park H, Kim J, Choi E. Association between OH/ECW and echocardiographic parameters in CKD5 patients not undergoing dialysis. PLoS One. 2018;13: e0195202 10.1371/journal.pone.0195202 29630661 PMC5891010

[17] Horvath TL. The hardship of obesity: a soft-wired hypothalamus. Nat Neurosci. 2005;8: 561–565. 10.1038/nn1453 15856063

[18] Cone RD. Anatomy and regulation of the central melanocortin system. Nat Neurosci. 2005;8: 571–578. 10.1038/nn1455 15856065

[19] Cumin F, Baum HP, Levens N. Mechanism of leptin removal from the circulation by the kidney. J Endocrinol. 1997;155: 577–585. 9488003 10.1677/joe.0.1550577

[20] Beberashvili I, Sinuani I, Azar A, Yasur H, Feldman L, Efrati S, et al Nutritional and Inflammatory Status of Hemodialysis Patients in Relation to Their Body Mass Index. J Ren Nutr. 2009;19: 238–247. 10.1053/j.jrn.2008.11.007 19243974

[21] Aguilera A, Bajo M, Rebollo F, Díez JJ, Díaz C, Paiva A, et al Leptin as a marker of nutrition and cardiovascular risk in peritoneal dialysis patients. Adv Perit Dial. 2002;18: 212–217. 12402621

[22] Hung SC, Tung TY, Yang CS, Tarng DC. High-calorie supplementation increases serum leptin levels and improves response to rHuEPO in long-term hemodialysis patients. Am J Kidney Dis. 2005;45: 1073–1083. 15957137 10.1053/j.ajkd.2005.02.020

[23] Rammohan M, Kalantar-Zadeh K, Liang A, Ghossein C. Megestrol acetate in a moderate dose for the treatment of malnutrition-inflammation complex in maintenance dialysis patients. J Ren Nutr. 2005;15: 345–355. 10.1016/j.jrn.2004.10.006 16007564

[24] Maffei M, Fei H, Lee GH, Dani C, Leroy P, Zhang YY, et al Increased expression in adipocytes of ob RNA in mice with lesions of the hypothalamus and with mutations at the db locus. Proc Natl Acad Sci U S A. 1995;92: 6957–6960. 10.1073/pnas.92.15.6957 7624352 PMC41450

[25] Mak RH, Cheung W, Cone RD, Marks DL. Leptin and inflammation-associated cachexia in chronic kidney disease. Kidney Int. 2006;69: 794–797. 10.1038/sj.ki.5000182 16518340

[26] Knobelspies H, Zeidler J, Hekerman P, Bamberg-Lemper S, Becker W. Mechanism of attenuation of leptin signaling under chronic ligand stimulation. BMC Biochem. 2010;11: 2 10.1186/1471-2091-11-2 20059770 PMC2821298

[27] Nakazato M, Murakami N, Date Y, Kojima M, Matsuo H, Kangawa K, et al A role for ghrelin in the central regulation of feeding. Nature. 2001;409: 194–198. 10.1038/35051587 11196643

[28] Mafra D, Guebre-Egziabher F, Fouque D. Endocrine role of stomach in appetite regulation in chronic kidney disease: about ghrelin and obestatin. J Ren Nutr. 2010;20: 68–73. 10.1053/j.jrn.2009.08.002 19913441

[29] Gunta SS, Mak RH. Ghrelin and leptin pathophysiology in chronic kidney disease. Pediatr Nephrol. 2013;28: 611–616. 10.1007/s00467-012-2380-9 23229444

[30] Liu J, Prudom CE, Nass R, Pezzoli SS, Oliveri MC, Johnson ML, et al Novel ghrelin assays provide evidence for independent regulation of ghrelin acylation and secretion in healthy young men. J Clin Endocrinol Metab. 2008;93: 1980–1987. 10.1210/jc.2007-2235 18349056 PMC2386282

[31] Mafra D, Guebre-Egziabher F, Cleaud C, Arkouche W, Mialon A, Drai J, et al Obestatin and ghrelin interplay in hemodialysis patients. Nutrition. 2010;26: 1100–1104. 10.1016/j.nut.2009.09.003 20018486

